# Quantitative Lipidomic Analysis of Osteosarcoma Cell-Derived Products by UHPLC-MS/MS

**DOI:** 10.3390/biom10091302

**Published:** 2020-09-09

**Authors:** Sara Casati, Chiara Giannasi, Mauro Minoli, Stefania Niada, Alessandro Ravelli, Ilaria Angeli, Veronica Mergenthaler, Roberta Ottria, Pierangela Ciuffreda, Marica Orioli, Anna T. Brini

**Affiliations:** 1 Dipartimento di Scienze Biomediche, Chirurgiche ed Odontoiatriche, Università degli studi di Milano, 20133 Milan, Italy; mauro.minoli@unimi.it (M.M.); alessandro.ravelli@unimi.it (A.R.); ilaria.angeli@unimi.it (I.A.); veronica.mergenthaler@gmail.com (V.M.); marica.orioli@unimi.it (M.O.); anna.brini@unimi.it (A.T.B.); 2 IRCCS Istituto Ortopedico Galeazzi, 20161 Milan, Italy; chiara.giannasi@grupposandonato.it (C.G.); stefania.niada@grupposandonato.it (S.N.); 3 Dipartimento di Scienze Biomediche e Cliniche “L.Sacco”, Università degli studi di Milano, 20157 Milan, Italy; roberta.ottria@unimi.it (R.O.); pierangela.ciuffreda@unimi.it (P.C.)

**Keywords:** polyunsaturated fatty acids (PUFAs), eicosanoids, endocannabinoids, *N*-acylethanolamides, lipidomics, mass spectrometry, osteosarcoma

## Abstract

Changes in lipid metabolism are involved in several pathological conditions, such as cancer. Among lipids, eicosanoids are potent inflammatory mediators, synthesized from polyunsaturated fatty acids (PUFAs), which coexist with other lipid-derived ones, including endocannabinoids (ECs) and *N*-acylethanolamides (NAEs). In this work, a bioanalytical assay for 12 PUFAs/eicosanoids and 20 ECs/NAEs in cell culture medium and human biofluids was validated over a linear range of 0.1–2.5 ng/mL. A fast pretreatment method consisting of protein precipitation with acetonitrile followed by a double step liquid–liquid extraction was developed. The final extracts were injected onto a Kinetex ultra-high-performance liquid chromatography (UHPLC) XB-C18 column with a gradient elution of 0.1% formic acid in water and methanol/acetonitrile (5:1; *v*/*v*) mobile phase. Chromatographic separation was followed by detection with a triple-quadrupole mass spectrometer operating both in positive and negative ion-mode. A full validation was carried out in a small amount of cell culture medium and then applied to osteosarcoma cell-derived products. To the best of our knowledge, this is the first lipid profiling of bone tumor cell lines (SaOS-2 and MG-63) and their secretome. Our method was also partially validated in other biological matrices, such as serum and urine, ensuring its broad applicability as a powerful tool for lipidomic translational research.

## 1. Introduction

### 1.1. Bioactive Lipids

Bioactive lipids comprise a variety of molecules, whose biosynthesis and activity are responsible for several cell functions, including cell membrane integrity, energy storage, and lipid signaling, by exchanges within and outside the cell [1,2]. The biosynthesis of many lipids depends on the presence of their precursors, and changes in lipid metabolism are involved both in physiological processes and in pathological conditions, such as inflammation, immune system diseases, and cancer [1,2,3,4,5]. Moreover, lipid moieties are necessary for the generation of lipid messengers, such as arachidonic acid (AA)-derived eicosanoids, endocannabinoids (ECs), and long-chain fatty acid derivatives such as *N*-acylethanolamides (NAEs), which modulate important cellular processes, such as proliferation, apoptosis, and inflammation. Among these, eicosanoids are potent lipid inflammatory mediators, synthesized from polyunsaturated fatty acids (PUFAs) via cyclooxygenase (COX), lipoxygenase (LOX), and cytochrome P450. Prostaglandins, the first identified eicosanoids, are synthesized from AA by both COX-1 and 2. Cyclooxygenase-1 is constitutively expressed in almost all tissues, whereas COX-2 expression is mainly correlated to acute inflammation [6]. Prostaglandins (PGs) are pro-inflammatory molecules that promote the early stages of acute inflammation and are also implicated in the initiation and propagation of cancer [1,7]. Another component of the eicosanoids family is thromboxane, responsible for platelet aggregation and vascular smooth muscle contraction [8]. Alternatively, free AA may be metabolized by LOXs yielding leukotrienes, lipoxins, hepoxilins, and hydroxyeicosatetraenoic acids [9,10,11]. Moreover, PUFAs and eicosanoids exist in a dynamic balance with other different lipid-derived mediators, including ECs and NAEs [12,13]. Endocannabinoids are a family of lipid mediators obtained from long-chain PUFAs linked to amides, esters, or ethers able to modulate physiological responses through interaction with the endogenous cannabinoid system (ECS) [14]. The ECS is composed of lipid-derived ECs, their G-protein-coupled receptors (CB1 and CB2), and the enzymes responsible for their synthesis, transport, and metabolism [15]. The most common ECs, *N*-arachidonoylethanolamine (AEA) [16] and 2-arachidonoylglycerol (2AG) [17,18], are two AA-derivatives belonging to the large families of *N*-acylethanolamines and 2-monoacylglycerols, respectively. Since AEA and 2AG are both derivatives of AA, there is an intimate interrelationship between the EC and eicosanoid signaling systems. Other ECs have been identified, including *O*-arachidonoylethanolamine and *N*-arachidonoyldopamine (ADA), which are derived from non-oxidative metabolism of arachidonic acid [14]. Endogenous lipoamino acid analogs of AEA, including glycine (AGly), alanine, and serine (ASer) have been identified in mammals [19]. Moreover, ethanolamides and amino acids derivatives of long-chain saturated or polyunsaturated fatty acids, such as *N*-palmitoylethanolamide (PEA) and *N*-oleylethanolamide (OEA), belonging to the NAEs family, have been demonstrated to interact with the ECS components, leading to entourage effects [20,21]. Endogenous cannabinoid system ligands mediate most of the biological effects through their interactions with CB1 and CB2 receptors expressed in the central nervous system and on immune and peripheral cells [22]. Nevertheless, the ECs and NAEs interact not only with CB receptors but also with the deorphanized GPR55 receptors, transient receptor potential vanilloid 1 channel, and peroxisome proliferator-activated nuclear receptors that modulate anti-inflammatory and analgesic effects [23]. The deregulation of the ECs activity and the consequent alteration of the levels of the endogenous ECs and NAEs in different biological fluids have been associated to various pathological conditions [24,25], such as inflammation and pain perception [26]. It is clear that an altered qualitative or quantitative lipid profile, including PUFAs, eicosanoids, ECs, and NAEs, might be associated to pathological conditions and contribute to the outcome and progression of different pathologies. Therefore, a thorough understanding of the mechanisms underlying the action of PUFAs/eicosanoids and ECs/NAEs in bio-matrices requires a sensitive analytical method for an accurate identification and quantification of these molecules.

### 1.2. Lipid Analysis Features

Lipid analysis is challenging because of the very low concentrations in biological fluids and tissues (picograms to nanograms per milliliter or milligram), in vitro metabolism, and autoxidation. For the extraction of PUFAs/eicosanoids and ECs/NAEs from bio-matrices, an optimized solvent combination is necessary to cover the whole polarity and pKa ranges of these metabolites, including the polar prostaglandins and the less polar PUFAs. Several protocols for the extraction and the subsequent analysis of ECs and NAEs, mainly AEA and 2AG, or PUFAs and eicosanoids in various bio-matrices have been published [27,28,29]. The majority of liquid–liquid extraction (LLE) protocols according to Bligh and Dyer or Folch [30,31], are limited by the distribution of analytes in both water and chloroform layers. However, application of ternary solvent combinations including polar as well as nonpolar solvents seems to be a way to overcome these problems [28,29,32]. Moreover, the last step in sample preparation is often manual desalting by solid-phase extraction (SPE) [33,34]. Special requirements for lipid analysis in bio-matrices also include the preliminary deproteinization and pre-/post-extraction at reproducible temperature conditions. Finally, the limited amount of sample available from in vitro and preclinical studies should be taken into consideration since it may not be possible to perform multiple analyses.

### 1.3. Aim of the Work

In this case, we proposed a double LLE step from a single sample in different mixtures of organic solvents to cover a broad polarity range. The aim of the present work was to develop and validate fast and sensitive quantitative ultra-high-performance liquid chromatography-tandem mass spectrometry (UHPLC-MS/MS) methods using a simple LLE protocol for a simultaneous investigation of PUFAs, eicosanoids, and ECs and NAEs from small amounts of different bio-matrices. This new method, besides allowing a deep and quantitative lipid profiling of the four major lipid signaling families, has been validated in a wide range of different biological matrices, such as cell lysates, extracellular vesicles (EVs), conditioned medium (CM), urine, and serum. The validation of the method in very small amounts of these matrices ensures its applicability to a large number of different studies, leading to a powerful tool for lipidomic translational research.

### 1.4. Osteosarcoma-Derived Lipids

There is increasing evidence that the majority of ECS ligands exert significant effects on tumor cell growth, motility, spread, and metastasis rate [35,36,37,38]. In particular, in this work, we assess the lipid quantitative profile in osteosarcoma (OS)-derived samples. It has been demonstrated that the ECS influence bone cell activity and bone remodeling in physiological and pathological conditions such as cancer [39,40]. The most frequent primary cancers affecting skeletal system are osteosarcoma (OS) and chondrosarcoma [41]. In particular, OS is the most common malignant tumor of bone in children and young adults, exhibiting high invasion and metastasis rate [42]. It is well known that cancer cells may communicate via the release of soluble factors or EVs that are enriched not only in protein and nucleic acids but also in lipids. Several studies show a direct connection between tumor progression and inflammatory status [43,44]. Therefore, elucidating the lipidomic profile, in OS cells, OS-derived EVs, and secretome, might improve our understanding about OS biology. The OS-derived samples (cell lysates, CM, and EVs) were collected from Saos-2 and MG-63 cell lines, and a partial elucidation of their lipid composition was obtained. These results represent the first step in the challenging final aim of investigating the role of lipid signaling molecules in the crosstalk between OS and the surrounding microenvironment.

## 2. Material and Methods

### 2.1. Chemicals

Ultrapure water, acetonitrile, dichloromethane, isopropanol, methanol, ethyl acetate, n-exane, and hydrochloride acid were of analytical grade and purchased from Carlo Erba (Milan, Italy). Formic acid (98–100%) was purchased from Sigma–Aldrich (Milan, Italy). The reference materials *N*-arachidonoylethanolamide (AEA), *N*-linolenoylethanolamide (LNEA), *N*-linoleoylethanolamide (LEA), *N*-oleoylethanolamide (OEA), *N*-palmitoylethanolammide (PEA), *N*-stearoylethanolamide (SEA), and *N*-stearoylethanolamide-d4 (SEA-d4) were synthesized and completely characterized in our laboratories, as previously described [45,46]. The reference materials *N*-docosahexaenoylethanolamide (DHEA), *N*-eicosapentaenoylethanolamide (EPEA), *N*-arachidonoyldopamine (ADA), *N*-oleoyldopamine (ODA), *N*-arachidonoylglycine (AGly), *N*-oleoylglycine (OGly), *N*-palmitoylglicine (PalGly), *N*-arachidonoylserine (ASer), *N*-arachidonoylserotonine (A5HT), *N*-oleoylserotonine (O5HT), *N*-palmitoylserotonine (Pal5HT), 2-arachidonoylglycerylether (2AGE), 2-arachidonoyglycerol (2AG), *N*-arachidonoyl-3-hydroxy-γ-aminobutyric acid (AGABA), arachidonoyl acid (AA), eicosapentaenoyl acid (EPA), docosahexaenoic acid (DHA), thromboxane-B_2_ (TXB_2_), prostaglandin-F_2α_ (PGF_2α_), 6α-keto-prostaglandin-F1α (6α-keto-PGF_1α_), prostaglandin-E_2_ (PGE_2_), prostaglandin-D_2_ (PGD_2_), leukotriene-B4 (LTB_4_), 5-hydroxyeicosatetraenoic acid (5(*S*)-HETE), 15-hydroxyeicosatetraenoic acid (15(*S*)-HETE), and (±)14(15)-epoxyeicosatrienoic acid (14,15-EET), and internal standards *N*-arachidonoylethanolamide-d8 (AEA-d8), *N*-oleoylethanolamide-d2 (OEA-d2), *N*-palmitoylethanolammide-d5 (PEA-d5), *N*-eicosapentaenoylethanolamide-d4 (EPEA-d4), *N*-arachidonoyldopamine-d8 (ADA-d8), *N*-arachidonoylglycine-d8 (AGly-d8), *N*-arachidonoylserine-d8 (ASer-d8), *N*-oleoylserotonine-d17 (O5HT-d17), eicosapentaenoyl acid-d5 (EPA-d5), thromboxane-B_2_-d4 (TXB_2_-d4), prostaglandin-F_2α_-d4 (PGF_2α_-d4), and leukotriene-B_4_-d4 (LTB_4_-d4) were purchased from Cayman Chemical (Ann Arbor, MI, USA).

### 2.2. Cell Cultures

The Saos-2 and MG-63 cell lines (ATCC, Rockville, MD, USA) were plated in tissue culture vessels (Corning, New York, NY, USA) at a density of 5 × 10^3^ cells/cm^2^ in complete culture medium [47]: Dulbecco’s Modified Eagle Medium (DMEM, Euroclone, Milan, Italy) supplemented with 10% fetal bovine serum (Euroclone), penicillin 50 U/mL, 50 μg/mL streptomycin (Sigma Aldrich, Milan, Italy), and 2 mM L-glutamine (L-Glu, Euroclone). Cultures were maintained at 37 °C in a humidified atmosphere, containing 5% CO_2_. After 48 h culture, non-adherent cells were removed, and the medium replaced. At 70–80% confluence, the cells were detached with 0.5% trypsin/0.2% ethylenediaminetetraacetic acid (EDTA, Sigma Aldrich) and expanded.

### 2.3. Sample Collection

#### 2.3.1. Cell Samples

Once at 80–90% confluence, cells were washed twice with phosphate buffered saline (PBS, composed of NaCl 137 mM, KCl 2.7 mM, Na_2_HPO_4_ × 2H_2_O 8.1 mM, KH_2_PO_4_ 1.7 mM-pH 7.4) and kept for 1 h in starving medium (SM) (phenol red-free DMEM supplemented with 2 mM L-glutamine, 50 U/mL penicillin, 50 μg/mL streptomycin without fetal bovine serum) for additional washing. Medium was replaced by fresh SM and cells were starved for 72 h.

##### Concentrated Conditioned Media (CM)

Conditioned media were collected from approximately 6 × 10^6^ cells in starving conditions, centrifuged for 15 min at 2500× *g*, at 4 °C to remove debris and large apoptotic bodies, and concentrated through Amicon Ultra-15 Centrifugal Filter Devices with 3 kDa cut-off (Merck Millipore, Milan, Italy) for 90 min at 4000× *g*, 4 °C [48]. The final product was concentrated about 40–50 folds. The purified solution was analyzed for protein-anchored lipids or lipids enclosed in macromolecular components.

##### Extracellular Vesicles (EVs)

Extracellular vesicles were isolated from cell-conditioned medium using differential centrifugation, as previously described [49,50]. In brief, after 72 h of starvation, the conditioned medium from approximately 15 × 10^6^ cells was centrifuged for 15 min at 2500× *g*, 4 °C, and then ultra-centrifuged for 70 min at 100,000× *g* (L7–65; Rotor 55.2 Ti; Beckman Coulter, Brea, CA, USA) at 4 °C. Pellet was resuspended in sterile PBS and ultra-centrifuged again under the same conditions. The resulting EV pellet was kept at −20 °C for mass spectrometry analysis.

##### Cells Pellets

After 72 h of starvation, cells were harvested with 0.5% trypsin/0.2% EDTA and centrifuged for 4 min at 350× *g*. Cell pellets (approximately 1 × 10^6^ cells) obtained by this first centrifugation were washed twice with sterile PBS and stored at −20 °C until use.

#### 2.3.2. Serum and Urine Samples

Control human serum samples used for purification and extraction studies and for validation experiments were obtained from healthy volunteers, which gave informed consent to offer their biological samples for research intent. Blood samples were collected in Vacuette^®^ 6 mL non-gel serum separator tubes and aliquots of 1–2 mL serum were stored at −20 °C. Human urine specimens, obtained from volunteer colleagues, were collected after a circadian cycle and aliquots of 1–2 mL were stored at −20 °C until analysis.

### 2.4. Standard Solutions, Calibrators, and Quality Control (QC) Samples

Stock solutions of reference materials and internal standards (ISs) were prepared at the final concentration of 10 μg/mL by appropriate dilution with acetonitrile (ACN) under a stream of nitrogen. All solutions were stored in the dark at −20 °C. Working solutions were prepared in ACN from stock solutions and used for the preparation of calibration curves and quality QC samples at 100 ng/mL, except for AA, DHA, EPA, and EPA-d5 (1 μg/mL).

#### 2.4.1. Cell Samples

Calibration standards (CS) containing 0, 0.1, 0.25, 0.5, 1.25, 2.5, and 5 ng/mL for all compounds, 0, 1, 2.5, 5, 12.5, and 25 ng/mL for AA, DHA, and EPA, 1 ng/mL for ISs, and 10 ng/mL for EPA-d5 were prepared daily for each analytical batch by adding suitable amounts of working solutions to 500 μL of SM. Quality control samples were prepared in SM at three different concentration levels (low, intermediate, and high).

#### 2.4.2. Serum and Urine Samples

Calibrators and QC samples were prepared by adding ISs at the same concentration levels (see Section 2.4.1) to 500 μL of PBS, serum, and urine. Pooled serum and urine CS and QC used for validation experiments were prepared combining 20 and five different samples, respectively.

### 2.5. Sample Preparation

**Figure 1:**
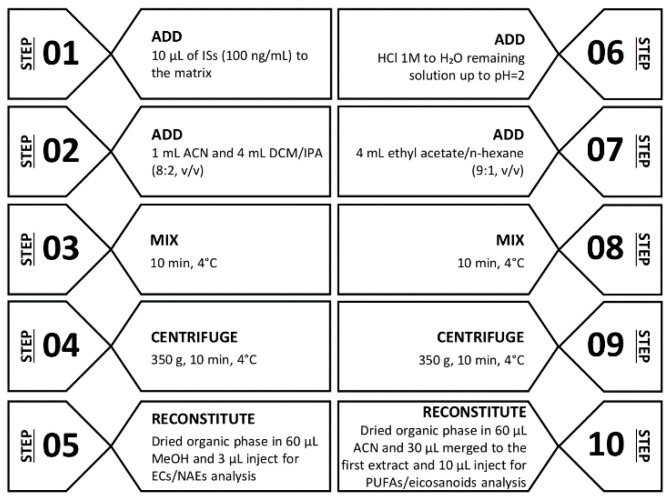
Diagram of the LLE procedure for ECs/NAEs (step 1–5) and PUFAs/eicosanoids (step 6–10). Abbreviations: internal standards (ISs), acetonitrile (ACN), dichloromethane/isopropanol (DCM/IPA), methanol (MeOH), endocannabinoids (ECs), *N*-acylethanolamides (NAEs), polyunsaturated fatty acids (PUFAs).

Extracellular vesicles and cell pellets, stored at −20 °C, were resuspended in 500 μL of SM and strongly vortexed three times for 1 min. Prior to extraction, 10 μL ISs and 1 mL of ice-cold ACN were added to 500 μL CM (as well as for serum and urine), EVs, and cell suspensions, and centrifuged for 10 min at 350× *g* at 4 °C. The clear supernatant was then transferred into glass test tubes and extracted with 4 mL of dichloromethane/isopropanol (8:2; *v*/*v*). After centrifugation at 350 g for 10 min, the organic layer was separated and dried under a stream of nitrogen. The dried residue was reconstituted with 60 μL methanol and a 3 μL aliquot was injected into the UHPLC-MS/MS system for ECs and NAEs analysis. The remaining aqueous solution was used for PUFAs and eicosanoids extraction, by adding 500 μL hydrochloride acid (HCl, 0.125 N) and 4 mL ethyl acetate/*n*-hexane (9:1; *v*/*v*). The organic phase was dried, and the residue was reconstituted with 60 μL ACN. A 30 μL aliquot of methanol obtained from the neutral extraction and a 30 μL aliquot from acid extraction were merged and transferred into an autosampler vial. A 10 μL aliquot was injected into the UHPLC/MS-MS system for PUFAs and eicosanoids determination (Figure 1).

### 2.6. Equipment

Analyses were performed on a 1290 Infinity UHPLC system (Agilent Technologies, Palo Alto, CA, USA) coupled to a Q Trap 5500 triple quadrupole linear ion trap mass spectrometer (Sciex, Darmstadt, Germany), equipped with an electrospray (ESI) source. Compounds were separated on a Kinetex UHPLC XB-C18 column (100 × 2.1 mm i.d, 2.6 p.s.) (Phenomenex, Torrance, CA, USA) using 0.1% formic acid in water (mobile phase A) and methanol/acetonitrile (5:1; *v*/*v*) (mobile phase B). For ECs and NAEs analysis, solvent A and B were 75% and 25% at 1.00 min, respectively. Solvent B was increased to 70% from 1.00 to 1.50 min, then increased to 85% from 1.50 to 6.00 min, and to 100% from 6.00 to 7.00, held at 100% from 7.00 to 9.00 min, and then decreased back to 25% from 9.00 to 9.20 min and held at 25% from 9.20 to 11.0 min for re-equilibration. For PUFAs and eicosanoids analysis, solvent A and B were 75% and 25% at 1.00 min, respectively. Solvent B was increased to 40% from 1.00 to 3.00 min, then increased to 95% from 3.00 to 5.50 min and to 100% from 5.50 to 7.00, held at 100% from 7.00 to 8.00 min, and then decreased back to 25% from 8.00 to 8.20 min and held at 25% from 8.20 to 10.0 min for re-equilibration. The flow rate was 0.60 mL/min and the column thermostatic oven was kept at 40 °C. The working conditions and parameters of the MS were optimized by direct infusion (flow rate 7 μL/min) of a standard mix solution (100 ng/mL) as follows: the ion source was ESI-operated in positive mode for the ECs/NAEs and in negative mode for PUFAs/eicosanoids analysis, resolution of Q1 and Q3 was 0.7 ± 0.1 amu (atomic mass unit), the curtain gas, ion gas 1, and ion source gas 2 were set at 25, 45, and 40 psi (pound per square inch) respectively, the source temperature was 550 °C, the ionization voltage was 5500 eV (positive mode) and −4500 eV (negative mode), the entrance potential was 10 eV, and dwell time was fixed 70 ms for each multiple reaction monitoring (MRM) transition. The MRM conditions and parameters including ion transitions, de-clustering potential (DP), and relative collision energy (CE) are provided in Table 1. In detail, the following product ions were applied:

- AEA, LNEA, LEA, PEA, OEA, SEA → *m*/*z* 62 relative to the protonated ethanolamine moiety.
- 2AG → *m*/*z* 287 relative to glycerol neutral loss.
- ODA, ADA → *m*/*z* 154 relative to the protonated dopamine moiety.
- A5HT, O5HT, Pal5HT → *m*/*z* 160 relative to the protonated dehydroxy-5HT moiety.
- ASer → *m*/*z* 106 relative to the protonated serine moiety.
- AGly, OGly, PalGly → *m*/*z* 76 relative to the protonated glycine moiety.

### 2.7. Data Evaluation

Data acquisition and processing were performed using Analyst^®^1.6.2 and MultiQuant^®^2.1.1 software (Sciex, Darmstadt, Germany), respectively. Calculations for validation assessment, which includes linearity, precision, accuracy, sensibility, recovery, and stability, were performed using Microsoft Office Excel 2013.

### 2.8. Validation Procedure

Assay validation was carried out in accordance with the recommendations endorsed by Food and Drugs Administration (FDA) guidelines referring to drugs and non-endogenous compounds [51], and specific issues for endogenous compounds [52] were addressed. A full validation was performed in the analyte-free SM and the following parameters were assessed: linearity, precision and accuracy, sensitivity in terms of limits of detection (LODs) and limits of quantitation (LOQs), specificity, recovery, matrix effect, and stability. Additionally, the described method was partially validated in serum and urine. Surrogate analyte-free matrix (i.e., water and/or appropriate buffer) are usually used for the preparation of CS and QC in the method validation of endogenous compounds to overcome the lack of analyte-free matrix [52]. For this reason, to avoid the interference of endogenous analytes, linearity, slope, recovery, and the influence of matrix effect were obtained by spiking serum and urine with ISs at the same concentration levels (see Section 2.4.2), whereas LOD and LOQ evaluation was achieved on PBS.

#### 2.8.1. Calibration Range and Linearity

Calibration standards (*n* = 6) were obtained by spiking analyte-free SM with appropriate amounts of working solutions in the range 0.1–2.5 and 0.5–25 ng/mL (EPA, AA, and DHA), as described at Section 2.4. A linear model was used to describe the relation between analyte concentration and instrument response (analyte peak area/internal standard peak area). Linearity was considered satisfactory for each curve if R² ≥ 0.990. Additionally, to evaluate linearity and slope, CS were also prepared in the analyte-free PBS, as well as in urine and serum, by spiking ISs at the same concentration levels.

#### 2.8.2. Sensitivity and Specificity

Reagents and consumables were extracted, following the procedures described before, and analyzed in triplicate to evaluate and exclude interferences and false-positive responses derived from sample preparation. The specificity of the method and matrix-to-matrix reproducibility was evaluated by analyzing SM in triplicate from different lots number (*n* = 3). Sensitivity was expressed in terms of LOD and LOQ as 3.3 and 10 times respectively, the ratio between the standard deviation of the response and the slope of the calibration curve. LOD and LOQ were calculated on calibration curves prepared in the analyte-free SM for cell samples’ quantification. Additionally, LOD and LOQ were also tested in the analyte-free PBS in order to quantify serum and urine samples.

#### 2.8.3. Precision and Accuracy

Precision and accuracy of the method were determined through the analysis of six independent replicates of QC materials extracted from the analyte-free SM at three concentration levels (low, intermediate, and high). Precision was denoted by percent coefficient of variation (CV%), while the accuracy was expressed as bias (BIAS%), the percent deviation of the mean determined concentration from the accepted reference value. The accuracy and precision were required to be ≤15% CV (Supplementary Tables S1 and S2).

#### 2.8.4. Recovery and Matrix Effects

Extraction recovery (%) was measured by comparing the peak area of the analyte-free SM (*n* = 3) fortified with standards at three concentration levels prior to and after extraction. Peak areas of pre- and post-extraction samples were used for calculations, considering as 100% recovery, the analytes area in post-extraction spiked samples. The matrix effects (%) were determined by comparing the analytes peak area in PBS and in the analyte-free SM, fortified in the low, intermediate, and high concentration range after extraction. Concerning the extraction recovery evaluation in human and serum and urine, which are matrices endogenously containing all the analytes, we spiked them with ISs before and after LLE. The matrix effect was assessed by comparing the peak area of ISs spiked in eluate from serum and urine to those in PBS. As for SM, the extraction recovery and matrix effect were evaluated at the three concentration levels.

#### 2.8.5. Stability Studies

Lipids’ stability was assessed in QC samples at low, intermediate, and high concentrations, by analyzing them the initial day (T0) as well as 24 h later at 4 and –20 °C. The response factor at each concentration was compared to the original vial at T0, and a mean deviation % below 15% from day 0 was considered acceptable.

### 2.9. Application to Real Samples

The proposed method was applied to Saos-2- and MG-63-derived CM, EVs, and cell lysates in order to identify and quantify lipids belonging to PUFAs/eicosanoids and ECs/NAEs groups, as described at Section 2.3. Each sample was injected into UHPLC-MS/MS three times (*n* = 3 analytical replicates).

## 3. Results and Discussion

### 3.1. Sample Extraction

Different protocols for the purification and several combinations of solvents over the expected polarity range were examined for the extraction of the considered analytes; however, the highest lipids count was detected through a double-step extraction preparation with dichloromethane/isopropanol (8:2; *v*/*v*) and ethyl acetate/n-hexane (9:1; *v*/*v*), respectively. On the basis of the analytes’ lipophilicity, a LLE procedure was developed with water-immiscible solvents in order to isolate both PUFAs/eicosanoids and ECs/NAEs. Despite the fact that in several studies SPE has provided concentrated and free interfering matrix components’ extracts [27,33,53,54], this extraction procedure is money- and time-consuming (because of the different steps). Contrarily, LLE is easier, and its shorter extraction time, as opposed to the most commonly used SPE procedures, could be an advantage for studies that involve a huge number of samples. LLEs commonly used to isolate lipids from biological samples require the use of toxic organic solvents. In our applied extraction protocol, a simple and fast pretreatment method consisting of protein exclusion with can, followed by a first extraction with dichloromethane/isopropanol (8:2; *v*/*v*) and a second one with ethyl acetate/n-hexane (9:1; *v*/*v*), both less toxic than other solvents (i.e., toluene, chloroform or tert-methyl-butyl ether), was used. In general, the combination of two or more sample preparation techniques, such as protein precipitation and LLE, improves method selectivity [29,53,54]. Additionally, the second extraction is preceded by a pH adjustment step, which is fundamental since some eicosanoids present a lower pKa value than ECs. In detail, acidification with HCl improves the extraction of the less polar eicosanoids HETEs and EETs. A lower pH leads to a reduced protein binding and the protonation of carboxylate anions, which both allow improved extraction by the organic solvent. Otherwise, greater acidification may lead to eicosanoid alteration [55], and therefore, an extremely low pH should be avoided. The optimized solvent mixture combined with the pH adjustment, which allows the decreased protein binding and the enhanced extraction of the non-ionized forms, was necessary to cover the whole polarity range of these numerous metabolites. Moreover, the two sequential extraction steps from a single sample also allowed the analysis when limited amounts of samples were available.

### 3.2. Instrumental Parameters

Mass spectrometry parameters were optimized by infusing a standard mix solution containing PUFAs, eicosanoids, ECs, and NAEs at a concentration of 100 ng/mL in methanol, and by acquiring both in the positive and negative ionization mode. Positive ionization mode provided better signal responses for the ECs/NAEs group, whereas for the analysis of PUFAs and eicosanoids, a negative polarization was used. The source/gas parameters were optimized to obtain the highest ion abundance of the peaks. CE and DP were varied from 0 to ±60 eV and 0 to ±150 eV respectively, in order to obtain the best response for the product ions used for quantitative MRM analysis. Precursors and product ions, CE and DP, shown in Table 1, were selected for analytes’ quantification. Several reversed phase columns, mobile phases, and elution gradients have been assessed in order to improve the responses of the target compounds belonging to EC/NAE and PUFA/eicosanoid classes and to reduce the time of the analysis. Two different elution gradients performed on a Kinetex UHPLC XB-C18 column using 0.1% formic acid in water and methanol/acetonitrile (5:1; *v*/*v*), characterized by a total runtime of 11.0 min each comprising cleaning and reconditioning of the column, exhibited the best analytes’ sensitivity and peak shape. These instrumental conditions allowed the consequential analysis of the two classes of interest using both the same elution column and mobile phases.

### 3.3. Method Validation

Methods specificity was achieved by means of the selection of a precursor ion followed by detection and quantification of product ions. All the reagents and consumables used for the methods set-up and development have been shown not to interfere with the detection or quantification of the analytes. False-positive response or co-eluting components were not detected in analyzed bio-matrices.

#### 3.3.1. Calibration Range and Linearity

Standard calibration curves (*n* = 6) were obtained by fortifying 500 μL aliquots of analyte-free SM with standard solutions, as described at Section 2.4.1. The calibration curves showed excellent linearity (R² > 0.991) over the following concentration ranges: 0.1–2.5 ng/mL for all the compounds and 1–25 ng/mL for AA, EPA, and DHA. Different calibration ranges for the eicosanoids AA, EPA, and DHA have been chosen in relation to expected higher concentrations in real samples. The R² values relative to PUFAs/eicosanoids and ECs/NAEs are reported in Table 2 and Table 3, respectively. Linearity was also maintained in all matrices assessed. Calibration curves prepared spiking ISs at the same concentration levels in PBS and in human serum and urine (see Section 2.4.2) were found to be parallel (standard deviation of correlation coefficients <0.0001). For this reason, calibration lines obtained from CS spiked in PBS may be used for PUFAs, eicosanoids, ECs, and NAEs quantification. Specificity tests, performed on all reagents and disposable materials used, have shown no interference with the determination of both PUFAs/eicosanoids and ECs/NAEs by UHPLC-MS/MS. LOD and LOQ values, obtained for the two lipid groups both in the analyte-free SM and PBS, are listed in Table 2 and Table 3.

#### 3.3.2. Precision and Accuracy

**Figure 2:**
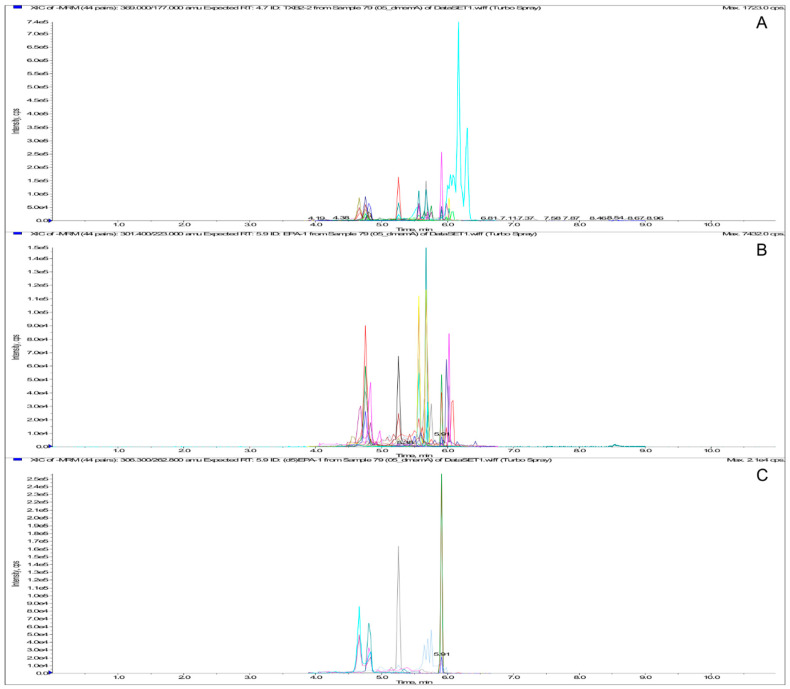
MRM chromatogram of PUFAs/eicosanoids extract. From the top: Total ion Current (**A**), standard extraction (0.5 and 5 ng/mL for AA, EPA, and DHA) (**B**), and ISs extraction (**C**).

**Figure 3:**
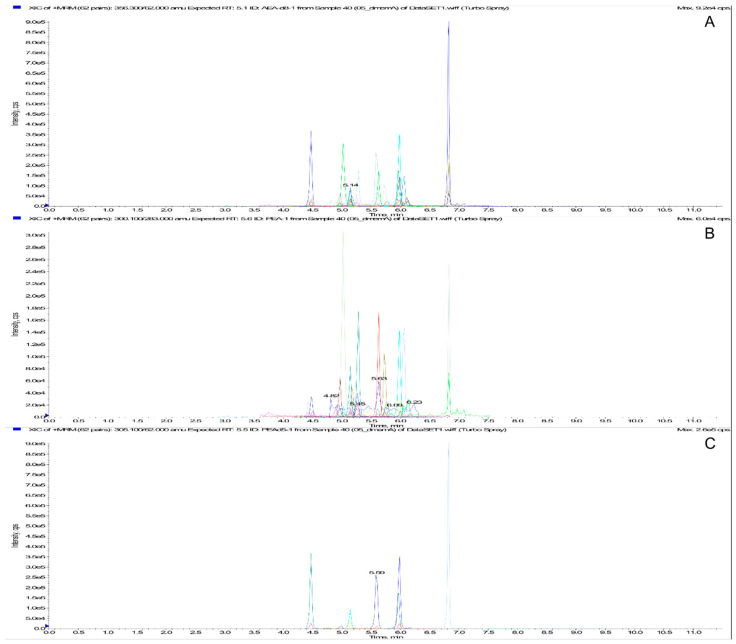
MRM chromatogram of ECs/NAEs extract. From the top: Total ion Current (**A**), standard extraction (0.5 ng/mL) (**B**), and ISs extraction (**C**).

Regarding precision and accuracy, the method showed good performance in terms of both repeatability and reproducibility, showing CV values below 15%. The same results were obtained for accuracy studies (BIAS < 15%). Precision and accuracy levels for all the analytes belonging to PUFAs/eicosanoids and ECs/NAEs groups were within acceptable limits, as reported in the Supplementary Tables S1 and S2, respectively. A representative chromatogram of a SM sample spiked at the intermediate concentration level for PUFAs/eicosanoids and ECs/NAEs groups is reported in Figure 2 and Figure 3, respectively.

#### 3.3.3. Extraction Recovery and Matrix Effect

**Figure 4:**
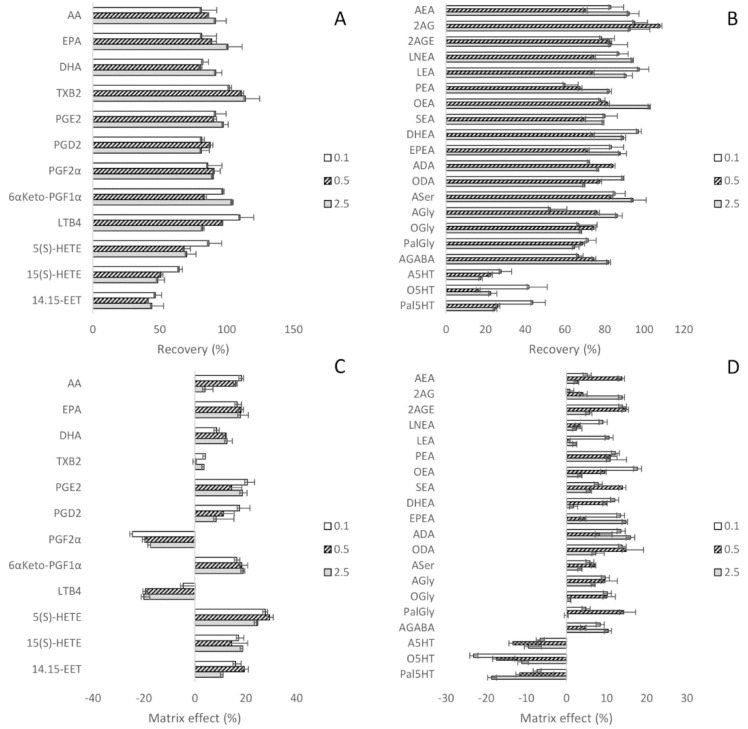
Extraction recovery and matrix effect of PUFAs/eicosanoids (**A**,**C**, respectively) and ECs/NAEs (**B**,**D**, respectively).

The mean extraction recovery in analyte-free SM was satisfactory, being over 41% for all the compounds belonging to the PUFAs/eicosanoids class (Figure 4A) and 52% for the ECs/NAEs class (Figure 4B), except for the basic compounds A5HT, O5HT, and Pal5HT. According to their chemical-physical properties, the 5HT-derivatives are protonated at neutral pH and the passage from aqueous solution to organic solvent is less-favored. Matrix effects ranged from ±20% for both lipids groups, except for PGF2α, 5(S)-HETE, and O5HT (Figure 4C,D). To avoid the interference of serum and urine endogenous analytes on the evaluation of recovery and matrix effect, the peak area of ISs spiked in these eluates was compared to those in the extract and PBS, respectively. Results for PUFAs/eicosanoids and ECs/NAEs are shown in the Supplementary Tables S3 and S4, respectively. All results were within the acceptance criteria, except for the PEA-d5, OEA-d2, and AGly-d8 matrix effect in human serum, whose percentage mean was 59% ± 8%, and TXB_2_-d4 in both serum and urine, which was 56% ± 7%.

#### 3.3.4. Stability Studies

The analytes’ concentration in QC samples was not altered when kept at 4 and –20 °C for 24 h, except for PGD_2_, 5(S)-HETE¸15(S)-HETE, and 14,15-EET, especially at –20 °C (Supplementary Table S5). The response factor did not show unacceptable differences compared with the first determination (mean deviation % from day 0 < 15%).

### 3.4. Application to Real Samples

**Figure 5:**
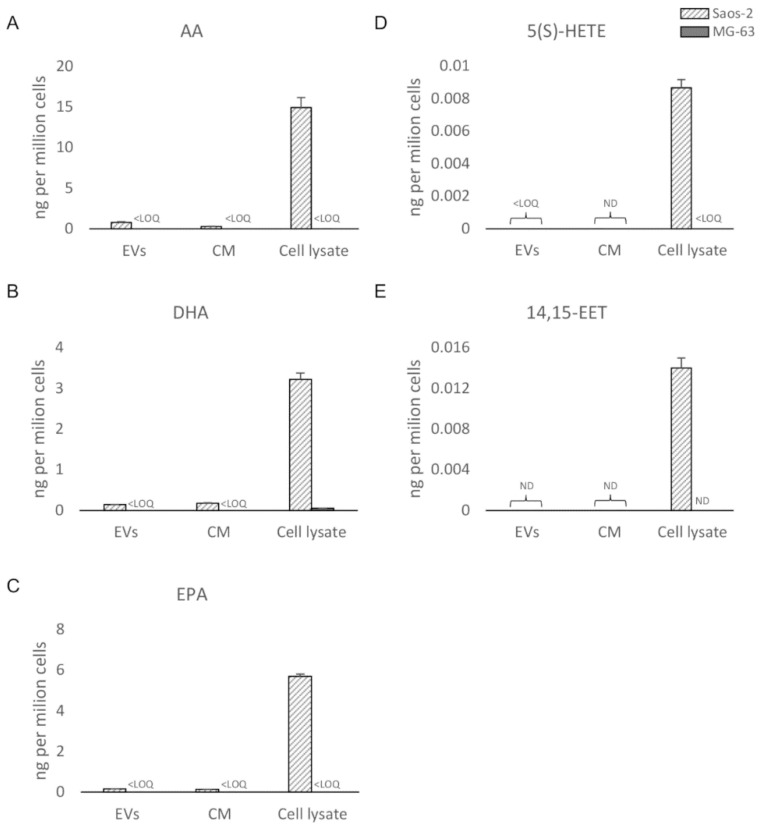
UHPLC-MS/MS quantitation (ND = not detectable; LOQ = limit of quantitation) in EVs, CM, and cell lysate from Saos-2 and MG-63 cell lines of (**A**) arachidonoyl acid (AA); (**B**) Docosahexaenoic acid (DHA); (**C**) eicosapentaenoyl acid (EPA); (**D**) 5-hydroxyeicosatetraenoic acid (5(*S*)-HETE; (**E**) (±)14(15)-epoxyeicosatrienoic acid (14,15-EET).

**Figure 6:**
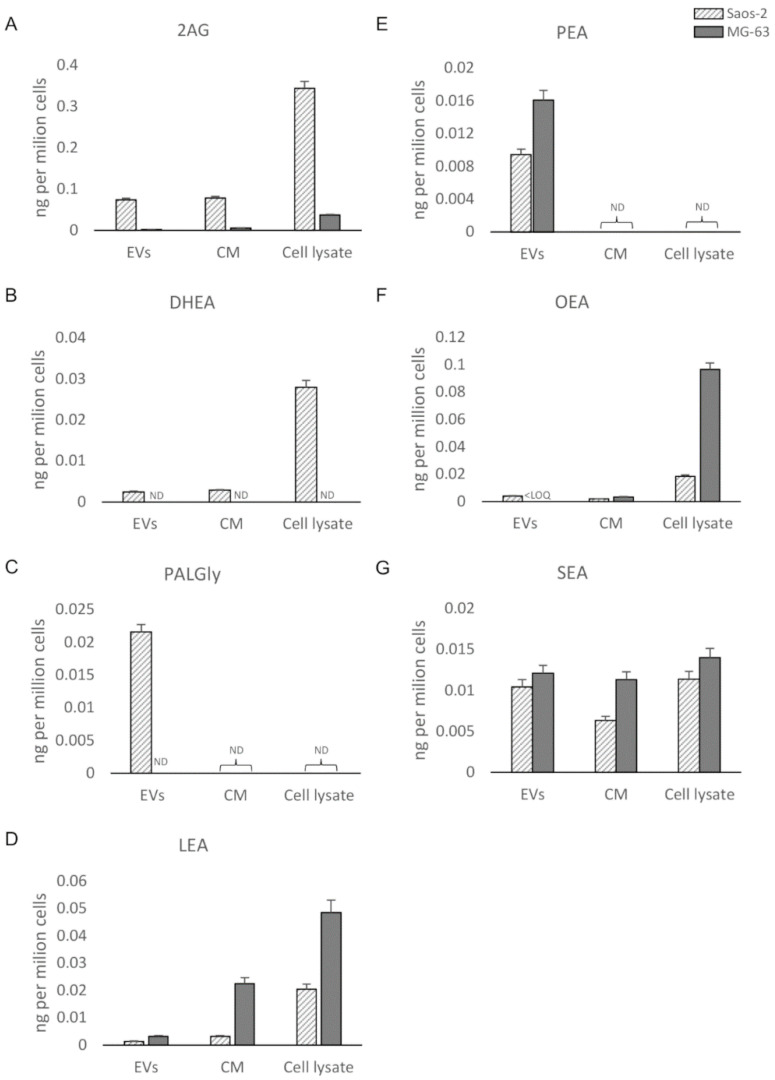
UHPLC-MS/MS quantitation (ND = not detectable; LOQ = limit of quantitation) in EVs, CM, and cell lysate from Saos-2 and MG-63 cell lines of (**A**) 2-arachidonoylglycerol (2AG); (**B**) *N*-docosahexaenoylethanolamide (DHEA); (**C**) *N*-palmitoylglicine (PalGly); (**D**) *N*-linoleoylethanolamide (LEA); (**E**) *N*-palmitoylethanolammide (PEA); (**F**) *N*-oleoylethanolamide (OEA); (**G**) *N*-stearoylethanolamide (SEA).

The bioanalytical assay was applied to osteosarcoma cell (Saos-2 and MG-63) lysates, EVs, and CM, as described at Section 2.3. By analyzing the CM, only protein-anchored lipids or lipids enclosed in macromolecular components were detectable. The filtered solution, accounting for the free lipid portion, will be analyzed in the near future. Quantitative data regarding the PUFAs/eicosanoids and ECs/NAEs in the six samples are presented in Figure 5 and Figure 6, respectively. Five PUFAs and eicosanoids (AA, EPA, DHA, 5(S)-HETE, and 14,15-EET) and seven ECs/NAEs (2AG, LEA, OEA, SEA, DHEA, PEA, and PalGly) were quantified (>LOQs). PUFAs (AA, DHA, and EPA) (Figure 5A–C) and AA-derived metabolites 5(*S*)-HETE, 14,15-EET, and 2AG (Figure 5A,D,E) were more expressed in Saos-derived samples than in MG-63-derived ones. Surprisingly, almost no PUFA/eicosanoid was detectable in MG-63 samples, except for a small amount of DHA only measured in the cell lysate (Figure 5B). Among ECs, PalGly is the only compound belonging to N-acylglycines, which was found only in Saos-2-derived EVs (Figure 6C). Only three ECs/NAEs (2AG, LEA, and SEA) were quantified in all six analyzed samples (Figure 6C,D,G). LEA, PEA, OEA, and SEA (Figure 6D–G) were found more abundant in MG-63-derived samples, with PEA detectable only in EVs (Figure 6E). Interestingly, 2AG and DHEA (Figure 6A,B) were more abundant and/or quantified only in Saos-2-derived samples, as well as their related compounds AA and DHA, respectively (Figure 5A,B). Several studies have focused on the differences in growth, gene expression, and immunohistochemical profiles of OS cell lines [56,57], revealing that they possess peculiar characteristics. In particular, Saos-2 cells exhibit a more mature osteoblastic phenotype with a stronger alkaline phosphatase activity and a greater expression of osteoblastic markers (osteocalcin, bone sialoprotein, decorin, and procollagen-I) than MG-63. The latter present both mature and immature osteoblastic features, with only a small subpopulation expressing the typical osteoblastic markers. Here, we provide evidence of several differences in the bioactive lipid profile between these two bone tumor cell lines and their derivatives (both whole secretome and isolated EVs). In this perspective, a recent work by Roy et al. [58] investigated the lipid profile of two OS cell lines (the nonmetastatic HOS (human osteosarcoma) and the metastatic 143B cells). The authors reported interesting differences in the expression of lipids involved in the metastatic process between the two OS cell lines and in tumorigenesis in comparison to normal feline osteoblasts (FOB).

## 4. Conclusions

In this work, a bioanalytical assay for 12 PUFAs/eicosanoids and 20 ECs/NAEs in culture medium, human serum, and urine was developed and validated over a linear range of 0.1–2.5 or 1–25 ng/mL (AA, EPA, and DHA). The method was fully validated in cell culture medium and partially in urine and serum. Our double-step LLE protocol was found to be suitable for the simultaneous investigation of PUFA, eicosanoid, ECs, and NAEs content by UHPLC–MS/MS in small amounts of bio-matrices. The protocol allows simultaneous and reproducible analyses of a broad range of chemically different bioactive lipids by a pH adjustment. The proposed protocol for cell lysates, EVs, and CM can be easily adapted to other liquid and/or solid bio-matrices. With the LLE technique, we have achieved a shorter extraction time, if compared to the most common SPE procedure, and this represent a clear advantage for studies that involve a huge number of samples; moreover, the two sequential extraction steps from a single sample also allow the analysis when limited amounts of samples are available. However, the limitations of the protocol may be the great manual effort and the lack of automation. In more detail, the validated method applied to OS cell lysates, EVs, and CM allowed the quantification of five eicosanoids and seven ECs/NAEs (>LOQs). Eicosanoids and ECs/NAEs are biologically active lipid mediators that play a critical role in different pathological processes, and little is still known about their release in secretome/EVs from OS cell lines. In this work, we investigated the lipid content of Saos-2, MG-63, and their derivatives, providing evidence of a different lipid profile and secretion between the two OS cell lines. This method could be harnessed to investigate other components of OS microenvironment, relevant for the cellular crosstalk among bone tumor cells, normal osteoblasts, and mesenchymal stem/stromal cells, which is actually investigated in our laboratory by a proteomic approach. Moreover, an all-encompassing profiling of the lipids expressed and secreted by OS cells in comparison to normal osteoblasts would provide an insight in the mechanisms of bone tumor development and eventually suggest potential therapeutic targets and/or new biomarkers for the diagnosis and monitoring of this pathology. These data could lay the basis to better elucidate the biological role played by lipid mediators in a pathological context, which will be investigated in the future by in vitro studies.

## Figures and Tables

**Table 1:** Multiple reaction monitoring (MRM) parameters: precursor and product ion transitions (quantifier underlined) for all the analytes and internal standards (ISs), de-clustering potential (DP), and collision energy (CE).

Compound	Precursor Ion (*m*/*z*)	Product Ions (*m*/*z*)	DP (eV)	CE (eV)
AA (20:4)	303.1	59.1	−45	−42
		259.6	−45	−20
EPA (20:5)	301.4	59.1	−55	−42
		203.1	−55	−20
DHA (22:6)	327.3	283.3	−80	−10
		59.1	−80	−35
TXB_2_	369	177	−50	−22
		195	−50	−20
PGE_2_	351.5	315	−50	−25
		271.1	−50	−25
PGD_2_	351.5	271	−50	−30
		189	−50	−30
PGF_2α_	353	291	−50	−35
		193	−50	−35
6αKeto-PGF_1α_	369.5	245	−50	−35
		163	−50	−35
LTB_4_	335	273	−45	−23
		195	−45	−23
5(S)-HETE	319.5	115	−50	−18
		301.1	−50	−18
15(S)-HETE	319.5	219	−50	−15
		301.2	−50	−15
14,15-EET	319.,5	219.1	−50	−22
		301	−50	−40
AEA	348	62	76	42
		133	76	33
2AG	379.4	287.3	76	18
		203	76	25
LNEA	322.3	62.2	85	35
		81.2	85	35
LEA	324.3	62.2	85	35
		109	85	32
PEA	300.1	62	98	19
		283	98	36
OEA	326.3	62.2	85	35
		309	85	21
SEA	328.3	62.2	85	35
		311.1	85	22
DHEA	372.3	62	85	18
		67	85	36
AGly	362.3	287	85	18
		76	85	18
ADA	440.5	137	95	34
		154	95	23
2AGE	365.3	273	85	10
		121	85	20
ODA	418.3	137	85	24
		154	85	35
EPEA	346.3	62	85	35
		135	85	35
ASer	392.5	106	85	35
		137.3	85	33
OGly	340.5	76	85	35
		265	85	35
PalGly	314.5	76	85	35
		239	85	20
AGABA	406.5	287.4	85	24
		84.1	85	55
A5HT	463.3	160.4	85	35
		132.2	85	35
O5HT	441.7	160.4	85	35
		132.2	85	35
Pal5HT	415.7	160.4	130	47
		132.2	130	47
TXB_2_-d4	373	199	−50	−22
		173	−50	−22
PGF_2a_-d4	357	295	−50	−35
		197	−50	−35
LTB_4_-d4	339	197	−45	−23
		277	−45	−23
EPA-d5	306.3	59.1	−50	−35
		208.1	−50	−18
AEA-d8	356.3	62	76	35
		70	76	35
SEA-d4	332.3	66.2	85	35
		62	85	18
EPEA-d4	350.3	66	85	35
		135	85	35
OEA-d2	328.3	62	85	35
		311	85	35
PEA-d5	305.1	62	85	35
		288	85	35
ADA-d8	448.5	137	85	35
		154	85	35
AGly-d8	370.6	76	85	20
		84	85	20
ASer-d8	400.6	106	85	35
		70	85	35
O5HT-d17	458.7	160.4	130	47
		132.2	130	47

Abbreviations: arachidonoyl acid (AA), eicosapentaenoyl acid (EPA), docosahexaenoic acid (DHA), thromboxane-B2 (TXB2), prostaglandin-F2α (PGF2α), 6α-keto-prostaglandin-F1α (6α-keto-PGF1α), prostaglandin-E2 (PGE2), prostaglandin-D2 (PGD2), leukotriene-B4 (LTB4), 5-hydroxyeicosatetraenoic acid (5(*S*)-HETE), 15-hydroxyeicosatetraenoic acid (15(*S*)-HETE), (±)14(15)-epoxyeicosatrienoic acid (14,15-EET), arachidonoylethanolamide (AEA), *N*-linolenoylethanolamide (LNEA), *N*-linoleoylethanolamide (LEA), *N*-oleoylethanolamide (OEA), *N*-palmitoylethanolammide (PEA), *N*-stearoylethanolamide (SEA), *N*-docosahexaenoylethanolamide (DHEA), *N*-eicosapentaenoylethanolamide (EPEA), *N*-arachidonoyldopamine (ADA), *N*-oleoyldopamine (ODA), *N*-arachidonoylglycine (AGly), *N*-oleoylglycine (OGly), *N*-palmitoylglicine (PalGly), *N*-arachidonoylserine (ASer), N-arachidonoylserotonine (A5HT), N-oleoylserotonine (O5HT), *N*-palmitoylserotonine (Pal5HT), 2-arachidonoylglycerylether (2AGE), 2-arachidonoyglycerol (2AG), *N*-arachidonoyl-3-hydroxy-γ-aminobutyric acid (AGABA), eicosapentaenoyl acid-d5 (EPA-d5), thromboxane-B2-d4 (TXB2-d4), prostaglandin-F2α-d4 (PGF2α-d4), and leukotriene-B4-d4 (LTB4-d4), *N*-arachidonoylethanolamide-d8 (AEA-d8), *N*-oleoylethanolamide-d2 (OEA-d2), *N*-palmitoylethanolammide-d5 (PEA-d5), *N*-stearoylethanolamide-d4 (SEA-d4), *N*-eicosapentaenoylethanolamide-d4 (EPEA-d4), *N*-arachidonoyldopamine-d8 (ADA-d8), *N*-arachidonoylglycine-d8 (AGly-d8), *N*-arachidonoylserine-d8 (ASer-d8), *N*-oleoylserotonine-d17 (O5HT-d17).

**Table 2:** Calibration parameters for PUFAs/eicosanoids group.

Compound	R^2^	Analytical Range (ng/mL)	LOD (SM) (ng/mL)	LOQ (SM) (ng/mL)	LOD (PBS) (ng/mL)	LOQ (PBS) (ng/mL)
AA (20:4)	1.000	1–25	0.259	0.864	0.014	0.046
EPA (20:5)	1.000	1–25	0.039	0.132	0.007	0.024
DHA (22:6)	0.999	1–25	0.013	0.042	0.022	0.073
TXB_2_	0.991	0.1–2.5	0.021	0.070	0.022	0.073
PGE_2_	1.000	0.1–2.5	0.018	0.061	0.010	0.035
PGD_2_	0.999	0.1–2.5	0.008	0.028	0.031	0.103
PGF_2α_	1.000	0.1–2.5	0.008	0.028	0.018	0.059
6aKeto-PGF_1α_	1.000	0.1–2.5	0.006	0.020	0.014	0.048
LTB_4_	0.999	0.1–2.5	0.011	0.037	0.033	0.110
5(S)-HETE	0.998	0.1–2.5	0.031	0.100	0.016	0.053
15(S)-HETE	0.999	0.1–2.5	0.012	0.041	0.021	0.070
14,15-EET	0.999	0.1–2.5	0.002	0.006	0.027	0.090

Abbreviations: arachidonoyl acid (AA), eicosapentaenoyl acid (EPA), docosahexaenoic acid (DHA), thromboxane-B2 (TXB2), prostaglandin-F2α (PGF2α), 6α-keto-prostaglandin-F1α (6α-keto-PGF1α), prostaglandin-E2 (PGE2), prostaglandin-D2 (PGD2), leukotriene-B4 (LTB4), 5-hydroxyeicosatetraenoic acid (5(*S*)-HETE), 15-hydroxyeicosatetraenoic acid (15(*S*)-HETE), (±)14(15)-epoxyeicosatrienoic acid (14,15-EET).

**Table 3:** Calibration parameters for ECs/NAEs group.

Compound	R^2^	Analytical Range (ng/mL)	LOD (SM) (ng/mL)	LOQ (SM) (ng/mL)	LOD (PBS) (ng/mL)	LOQ (PBS) (ng/mL)
AEA	0.9960	0.1–2.5	0.013	0.045	0.027	0.088
2AG	0.9918	0.1–2.5	0.004	0.015	0.027	0.089
2AGE	0.9986	0.1–2.5	0.008	0.026	0.015	0.049
LNEA	0.9965	0.1–2.5	0.033	0.109	0.019	0.064
LEA	0.9916	0.1–2.5	0.030	0.100	0.028	0.094
PEA	0.9954	0.1–2.5	0.030	0.101	0.027	0.090
OEA	0.9998	0.1–2.5	0.020	0.076	0.025	0.084
SEA	0.9999	0.1–2.5	0.005	0.018	0.013	0.045
DHEA	0.9964	0.1–2.5	0.020	0.081	0.028	0.092
EPEA	0.9982	0.1–2.5	0.010	0.033	0.005	0.017
ADA	0.9980	0.1–2.5	0.018	0.059	0.029	0.099
ODA	0.9998	0.1–2.5	0.031	0.106	0.029	0.099
ASer	0.9959	0.1–2.5	0.019	0.064	0.023	0.081
AGly	0.9998	0.1–2.5	0.030	0.101	0.029	0.099
OGly	0.9985	0.1–2.5	0.028	0.094	0.035	0.100
PalGly	0.9999	0.1–2.5	0.006	0.019	0.026	0.099
AGABA	0.9988	0.1–2.5	0.021	0.084	0.017	0.057
A5HT	0.9982	0.1–2.5	0.008	0.028	0.007	0.024
O5HT	0.9942	0.1–2.5	0.002	0.073	0.013	0.043
Pal5HT	0.9980	0.1–2.5	0.007	0.023	0.012	0.042

Abbreviations: arachidonoylethanolamide (AEA), *N*-linolenoylethanolamide (LNEA), *N*-linoleoylethanolamide (LEA), *N*-oleoylethanolamide (OEA), N-palmitoylethanolammide (PEA), *N*-stearoylethanolamide (SEA), *N*-docosahexaenoylethanolamide (DHEA), *N*-eicosapentaenoylethanolamide (EPEA), *N*-arachidonoyldopamine (ADA), *N*-oleoyldopamine (ODA), *N*-arachidonoylglycine (AGly), *N*-oleoylglycine (OGly), *N*-palmitoylglicine (PalGly), *N*-arachidonoylserine (ASer), *N*-arachidonoylserotonine (A5HT), *N*-oleoylserotonine (O5HT), *N*-palmitoylserotonine (Pal5HT), 2-arachidonoylglycerylether (2AGE), 2-arachidonoyglycerol (2AG), *N*-arachidonoyl-3-hydroxy-γ-aminobutyric acid (AGABA).

## Supplementary Materials

The following are available online at https://www.mdpi.com/2218-273X/10/9/1302/s1. Table S1: Precision and accuracy parameters for PUFAs/eicosanoids group, Table S2: Precision and accuracy parameters for ECs/NAEs group, Table S3: Extraction recovery and matrix effect of PUFAs/eicosanoids groups in human serum and urine, Table S4: Extraction recovery and matrix effect of ECs/NAEs groups in human serum and urine, Table S5: Stability of QC samples after exposure to 4 °C and – 20°C for 24 h.
